# The effect of early mobilisation (< 14 days) on pathophysiological and functional outcomes in animals with induced spinal cord injury: a systematic review with meta-analysis

**DOI:** 10.1186/s12868-024-00862-3

**Published:** 2024-03-25

**Authors:** Natalie Gray, Junaid Shaikh, Alison Cowley, Vicky Goosey-Tolfrey, Pip Logan, Nasir Quraishi, Vicky Booth

**Affiliations:** 1 School of Medicine, University of Nottingham, Nottingham, UK; 2 Nottingham University Hospitals NHS Trust, Nottingham, UK; 3 School of Sport, Exercise and Health Sciences, University of Loughborough, Loughborough, UK

**Keywords:** Mobilisation, Function, Pathophysiology, Meta-analysis

## Abstract

**Introduction:**

The optimum time to mobilise (standing, walking) following spinal cord injury (SCI) is unknown but may have implications for patient outcomes. There are no high-quality experimental studies that examine this issue, with a paucity of guidance for clinicians. Pre-clinical studies lead research in this field and can contribute to knowledge and support future clinical practice. Objective: to evaluate the effect of early compared to no mobilisation on pathophysiological and functional outcomes in animals with induced SCI.

**Methods:**

A systematic review with meta-analysis was conducted by searching pre-clinical literature in MEDLINE (PubMed), Embase (Ovid), Web of Science, OpenGrey, and EThOS (June 2023). Studies were included of any research method giving numerical results comparing pathophysiological and functional outcomes in rats and mice mobilised within 14-days of induced SCI to those that did not mobilise. Data were synthesised using random-effects meta-analyses. The quality of the evidence was assessed using the CAMARADES checklist. The certainty of findings was reported using the GRADE approach. This study is registered on PROSPERO (CRD42023437494).

**Results:**

Seventeen studies met the inclusion criteria. Outcomes found that Brain Derived Neurotrophic Factor levels were greater in those that initiated mobilisation within 14-days of SCI compared to the groups that did not. Mobilisation initiated within 14-days of SCI was also associated with statistically significant functional gains: (Basso, Beattie and Bresnahan locomotor rating score (BBB) = 2.13(0–21), CI 1.43, 2.84, Ladder Rung Walking Task = − 12.38(0–100), CI 20.01, − 4.76). Meta-analysis identified the greatest functional gains when mobilisation was initiated within 3 days of SCI (BBB = 3.00, CI 2.31–3.69, p < 0.001), or when delivered at low intensity (BBB = 2.88, CI 2.03–3.70, p < 0.001). Confidence in the findings from this review was low to moderate due to the risk of bias and mixed methodological quality.

**Conclusion:**

Mobilisation instigated within 14-days of injury, may be an effective way of improving functional outcomes in animal models following SCI, with delays potentially detrimental to recovery. Outcomes from this study support further research in this field to guide future clinical practice.

**Supplementary Information:**

The online version contains supplementary material available at 10.1186/s12868-024-00862-3.

## Introduction

Spinal cord injury (SCI) refers to damage to the spinal cord from trauma or disease and can impact on all aspects of an individual’s life [1]. Rehabilitation is beneficial in the facilitation of recovery of functional capacity and prevention of further disability [2–6]. This includes mobilisation, referring to movements such as standing or walking [7]. Benefits of mobilisation include postural control, strength, balance, circulation, and bladder and bowel function, leading to functional and psychological gains [7–10]. However, an initial period of immobilisation, such as bed rest of 2–6 weeks, is used in the UK following concerns that early mobilisation may exacerbate secondary pathophysiological responses (e.g. inflammation, oedema) and hence hinder functional recovery [6, 7, 11–13]. The role of immobilisation following SCI has caused debate, with many international centres mobilising within 24–48 h of injury. This has led to a lack of consensus on when to initiate mobilisation to optimise outcomes and variation in clinical practice [6, 9, 11, 13, 14]. This has implications for patient outcomes and cost effectiveness, with multiple recommendations for further investigation [11, 13, 15].

Current evidence in this area has offered little clarity with a lack of randomised control trials. There are reports of greater functional gains and reduced complications when mobilisation is instigated < 4 weeks (compared to > 4 weeks) from injury [12, 16, 17]. However, the minimal number and low quality of these studies suggest the current available research is not robust enough to give confidence or clarity to inform the optimum time to initiate mobilisation [12, 16, 17]. There remains a scarcity of evidence on outcomes following mobilisation initiated within 14-days of injury, nor an investigation of the impact of mobilisation on these secondary pathophysiological responses. The potential for future studies in this field remains limited due to the practical difficulties of a small and heterogenic population (injury levels, severity), and lack of standardised interventions of mobilisation (including intensity, frequency, duration) as well as a lack of agreement on clinical equipoise leading to ethical challenges, making a resolution challenging [6, 9, 16].

This has led to the decision to further knowledge by exploring pre-clinical animal studies reporting outcomes following mobilisation initiated within 14-days of injury, where clinical studies have not investigated [18–20]. This has the advantage of an available body of data reporting both pathophysiological and functional outcomes following interventions of mobilisation (e.g. treadmill training) initiated within this early period [18, 19]. Measures of pathophysiology are commonly used in pre-clinical research including levels of brain-derived neurotrophic factor (BDNF) as an indicator of neuroplasticity and neural recovery (with higher levels a marker of better recovery), and indicators of inflammation, e.g. numbers of microglial (with lower levels a marker of better recovery) [18, 21, 22]. A range of standardised and validated measures of function are also reported within these pre-clinical studies [3, 23, 24].

The pre-clinical literature has often reported favourable outcomes following early mobilisation, although contrasting findings exist and significant variables across the studies has made it difficult to compare or consolidate findings [18–20, 25]. A systematic review was therefore proposed to investigate the effect of early mobilisation on pathophysiological and functional outcomes in pre-clinical studies. This aimed to increase knowledge and understanding of the risks and benefits of mobilisation initiated within 14-days of SCI, with the potential for translation to support further research in this field and guide future clinical practice.

### Research question

What is the effect of early mobilisation (< 14 days) compared to no mobilisation on pathophysiological and functional outcomes in animals with induced SCI?

## Methods

The Preferred Reporting Items for Systematic Reviews and Meta-Analysis (PRISMA) guidelines were followed for this review [26]. The review protocol was registered in PROSPERO (CRD42023437494) [27].

### Eligibility criteria

#### Types of studies

This review considered all study types reporting on outcomes of mobilisation initiated within 14 days of injury in comparison to those not mobilised. Studies were not excluded based on publication year, geographical location, or language if a translated copy was available. Conference abstracts, secondary analysis and qualitative studies were excluded.

#### Types of participants

The review was limited to studies of rats and mice (all species, all sexes, all ages) with an experimentally induced SCI, including contusion, compression, hemi-section, and complete transection. This included injury at all spinal levels and any severity.

#### Types of interventions

The review considered literature that reported on any therapeutic intervention of mobilisation initiated within 14 days of SCI, such as walking or running using a treadmill, wheel or ball, or ladder climbing. Any intensity (e.g. high or low) and frequency (number of mobilisation sessions) was eligible for inclusion. Studies that reported on swimming were excluded due to reduced transferability into clinical practice during this early treatment period. Studies were also excluded that reported on electrical stimulation, acupuncture or combined interventions including the use of pharmaceuticals.

Studies were sought that included a comparator group where the animal received an experimentally induced injury to the spinal cord but was not mobilised, e.g. sedentary or not trained.

#### Types of outcomes

Studies were sought that included outcomes of pathophysiology or functional movement using the following outcome measures:

##### Pathophysiology

1. Brain derived neurotrophic factor (BDNF), expressed as a ratio or percentage [18, 21].

2. Number of microglia (immune cells of the central nervous system) as an indicator of inflammation [18, 25, 28].

Pathophysiological outcomes reported for other areas of the nervous system, such as the brain, were excluded.

##### Functional movement

1. Basso, Beattie and Bresnahan locomotor rating score (BBB), scored 0–21, as a standardised, valid and predictive measure of locomotion [23].

2. Ladder Rung Walking Task expressed as a percentage of foot faults, as a measure of agility and motor function [24].

### Search strategy

A search of the literature was conducted June-July 2023 to select published papers meeting the eligibility criteria. Key words and subject headings (e.g. MeSH) were used in the areas of “spinal cord injury”, “exercise”, “mobilisation”, “pathophysiological”, “function” and “animals”. The following databases were searched: MEDLINE (PubMed), Embase (Ovid), and Web of Science. Grey literature was also searched through OpenGrey and EThOS. An independent research librarian contributed to the search strategy. Terms and key words were used and adapted to reflect differences in spelling and unique search features of individual databases. Reference lists of relevant reviews and included studies were also searched to identify any further relevant studies. An example search strategy is found in Additional file 1.

### Study selection

Titles and abstracts of studies retrieved using the search strategy were screened independently in Rayyan to identify studies that met the inclusion criteria (in full by JS, 10% by NG). A further search of the full text was completed independently by two reviewers (JS, NG). Consensus was reached following discussion. Consultation with a third reviewer to resolve disagreements was not required.

### Data extraction

Relevant data was extracted from the included studies using a data extraction tool, which was previously trialled and amended as required (in full by JS, 10% by NG). Demographic and outcome data was extracted from the studies, including sample size, dose, and timing of mobilisation. Only data from sample groups relevant to the inclusion criteria were extracted.

Outcome data were extracted at 4 weeks post SCI to gain standardisation across studies and based on the mean point of plateau in recovery [18, 29]. Where data was not available at this point, studies were also included where weeks 3 and 5 were available and a mean score for outcome and variance was used. Where more than 1 group was presented in a study, outcomes were reported separately.

### Quality assessment

All included studies were assessed for methodological quality using the Collaborative Approach to Meta-Analysis and Review of Animal Data from Experimental Studies quality score checklist (0–9) (CAMARADES) [30]. The quality of the study was considered low if scored < 4, medium 4–6, and high if > 6. This was independently completed by two reviewers (JS, NG,).

### Data synthesis

Studies were assessed for suitability for meta-analysis where variance (SEM or SD) was reported. Meta-analysis was conducted using a random effects model [29]. This was also used to evaluate statistical heterogeneity using I^2^ index, and considered high when I^2^ > 60% [29]. Findings were presented as multiple groups if reported as such within the studies. Subgroup analysis was conducted based on timing and intensity of intervention, where sufficient data was available. Animal type, type of injury and type of intervention did not report sufficient data or significant variation within the studies for a viable sub-group analysis. Analysis of the studies were summarised using tables, forest plots and narrative description. Mean differences were presented using 95% confidence intervals and P-values, with p < 0.05 considered statistically significant. Data analyses were performed using Review Manager (version 5.4.1) [31].

### Risk of bias assessment

All included studies were assessed for risk of bias using the Systematic Review Centre for Laboratory animal Experimentation tool (SYRCLE), adapted from Cochranes’ risk of bias tool [32]. Each study was independently completed by two reviewers (JS, NG, VB, AC). Consensus was reached following discussion with a third reviewer as necessary. These assessments were used to evaluate the quality of evidence and impact of bias on the overall findings. Certainty of the body of evidence was assessed using the GRADE methodology [29, 33].

## Results

### Details of included studies

**Fig. 1:**
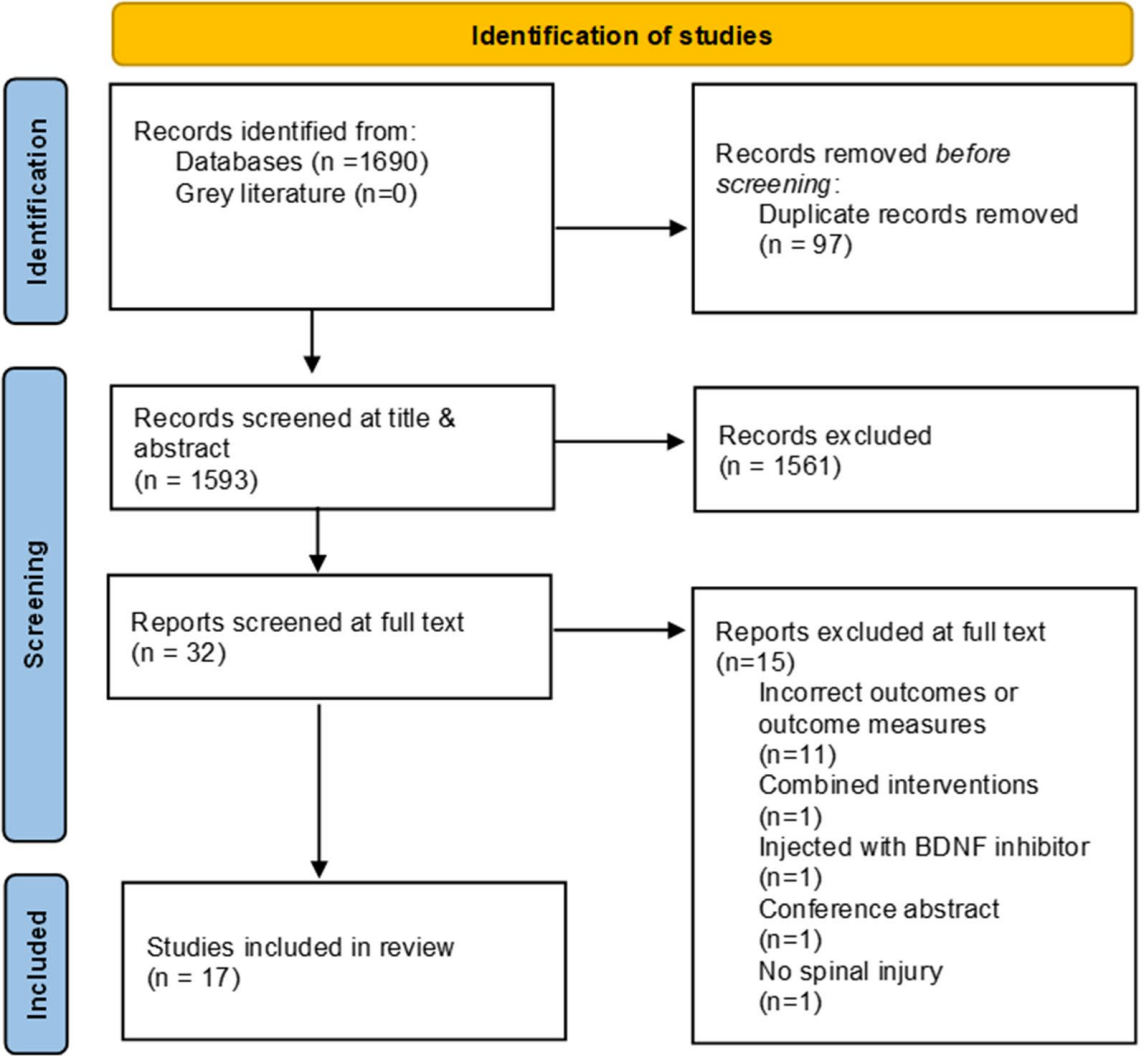
PRISMA flow diagram of search

17 studies were included in this review. Figure 1 shows the results of the search and selection process.

### Excluded studies

A list of studies excluded at full text stage and the reason for their exclusion is given in Additional file 2.

### Description of studies

Of the 17 included studies, seven reported pathophysiological outcomes [22, 34–39] and 15 reported functional outcomes [22, 37–49]. Characteristics of the 17 selected studies are presented in Table 1. Where more than one group was presented within a study, outcomes were reported separately.

**Table 1:** Characteristics of the included studies

Author & year	Country of study	Population					Intervention					Control	Time to mobilisation after SCI (days)	Outcome measure	
		Animal	Gender	Sample size	Type of injury	Injury level	Type	Duration* (minutes)	Frequency (per week)	Intensity (metres/min)	Duration of intervention period (to assessment)			Physiological	Functional
Asano 2022	Japan	Rat (W)	F	I = 6, C = 7	Transection	T10	Walking (wire mesh)	10	5	NR	3 weeks	No training	2	Microglial	BBB
Brown 2011 (a) (b)	USA	Rat (SD)	M	a) I = 6, C = 6 b) I = 6, C = 6	Contusion	T12	Exercise ball	NR	7	NR	2 weeks	No training	a) 1 b) 8	x	BBB
Cote 2011	USA	Rat (SD)	F	I = 22, C = 8	Transection	T12	Treadmill	15	5	NR	4 weeks	No training	5	BDNF	x
Davaa 2021	Korea	Rat (SD)	F	I = 14, C = 13	Contusion	T9	Treadmill	15 (× 2 day)	5	5–12	4 weeks	No training	7	x	BBB, Ladder
Han 2014	China	Rat (SD)	M	I = 18, C = 18	NR	T8-9	Rehabilitation training	30	3	NR	3 weeks	No training	3	x	BBB
Ilha 2011	Brazil	Rat (W)	M	I = 10, C = 10	Transection	T8-9	Treadmill (BWS)	20–30	5	6–7	4 weeks	No training	6	x	BBB
Li 2013 (a) (b) (c)	China	Rat (W)	M	a) I = 6, C = 6 b) I = 6, C = 6 c) I = 6, C = 6	Contusion	T10-11	Treadmill (BWS)	20	5	4.5	4 weeks	No training	a) 1 b) 2 c) 3	x	BBB
Loy 2018	Germany	Mice	F	I = 13, C = 13	Hemisection	T8	Exercise wheel	NR	NR	NR	4 weeks	No training	2	x	Ladder
Macias 2009	Poland	Rat (W)	M	I = 6, C = 6	Transection	T9-10	Treadmill	4 (× 4–6 day)	5	3	4 weeks	No training	7	BDNF	x
Marques 2018 (a) (b)	Brazil	Rat (W)	M	a) I = 13, C = 38 b) I = 14, C = 38	Contusion	T10	Treadmill	20	5	6	4 weeks	No training	a) 7 b) 14	BDNF	BBB, Ladder
Miranda 2012	Brazil	Rat (W)	M	I = 29, C = 11	Contusion	T9-10	Walking (runway)	12	2	NR	4 weeks	No training	3	x	BBB
Multon 2003	Belgium	Rat (W)	F	I = 7, C = 6	Compression	T9	Treadmill	10 (× 3 day)	5	3.5	4 weeks	No training	2–4	x	BBB
Oh 2009	Korea	Rat (SD)	M	I = 7, C = 7	Contusion	T9-10	Treadmill (BWS)	30	5	8	4 weeks	No training	7	x	BBB, Ladder
Sandrow-Feinberg 2009	USA	Rat (SD)	F	I = 8, C = 6	Contusion	C4	Exercise wheel	20	5	3.5–14	4 weeks	No training	5	BDNF	BBB, Ladder
Wang 2015	USA	Rat (SD)	F	I = 12, C = 12	Contusion	T9	Treadmill	15 (× 2 day)	5	5–12	4 weeks	No training	7	BDNF	BBB
Zhan 2023 (a) (b) (c)	China	Mice	F	a) I = 5, C = 15 b) I = 5, C = 15 c) I = 5, C = 15	Crush	C5	Treadmill	30	5	Starting 9, progressed to: a) ‘low’ b) ‘med’ c) ‘high’ (details NR)	4 weeks	No training	7	BDNF	Ladder
Zhao 2020 (a) (b) (c)	China	Rat (SD)	M	a) I = 10, C = 10 b) I = 10, C = 10 c) I = 10, C = 10	Contusion	T10	Treadmill (BWS)	30	5	4.2	3 weeks	No training	a) 3 b) 7 c) 14	x	BBB

SD (Sprague–Dawley), W(Wistar), M (male), F (female), I (intervention), C (control), BWS (body weight support), NR (not reported), BDNF (Brain derived neurotrophic factor), BBB (Basso, Beattie and Bresnahan locomotor rating score), x (outcome not measured). *Once a day unless otherwise stated

Papers reported samples ranging from n = 12–65 and consisted of male and female murids (M:F = 3:2) (Sprague–Dawley rats [34, 38–42, 44, 49], Wistar rats [22, 35, 36, 45–48], and mice [37, 43]) aged between 2 and 4 months. The studies used animal models with induced SCI resulting in complete SCI by transection [22, 34, 35, 48], or incomplete SCI by contusion [36, 38–41, 44, 45, 47, 49], hemi-Sect. [43] compression [46], or crush injury [37]. One study did not report the type of injury [42]. Two studies investigated animals with SCI at cervical level [37, 39] with all other studies reporting on animals with a thoracic injury (T8-12) [22, 34–36, 38, 40–49].

Mobilisation interventions comprised of treadmill training, including use of body weight support [34–38, 41, 44, 46–49], use of an exercise wheel or ball [39, 40, 43], walking on a runway [45] or wire mesh [22], or unspecified rehabilitation training [42]. Animals in the control groups were not given the opportunity to exercise; where reported this was described as ‘untrained’ or ‘sedentary’.

The training regime for most animals was 20–30 min per day, 5-days per week [35–39, 41, 44, 46–49], delivered as one session [36, 37, 39, 44, 47–49] or divided into multiple sessions throughout the day [35, 38, 41, 46]. Two studies delivered the intervention for 10–15 min once per day for 5-days per week [22, 34], one study delivered 30 min per day for 3-days per week [42], and one study for 12 min per day twice per week [45]. Two studies did not report the frequency or duration of the intervention [40, 43]. Intensity of exercise was reported as a measure of speed of the treadmill or wheel. This varied between 3.5 and 14 m/minute, with 3 studies gradually increasing the speed during the trial period depending on the capabilities of the animal [37, 39, 41]. Intensity of exercise was not reported in six studies [22, 34, 40, 42, 43, 45].

Mobilisation was initiated between 1 and 14 days following SCI. Three studies reported outcomes on groups initiating mobilisation at different time points within the 14 days [36, 40, 44]. Outcomes were extracted at 4 weeks post SCI. The interventions were delivered until this 4-week assessment point in almost all except 4 studies who delivered intervention up to 3-weeks [22, 42, 44] and 1 study up to 2-weeks [40].

### Quality assessment

All studies were assessed for methodological quality using CAMARADES checklist [30] (Table 2).

**Table 2:** Methodological quality assessment using CAMARADES checklist

	Publication in peer reviewed journal	Statement of control of temperature	Randomization of treatment or control	Allocation concealment	Blinded assessment of outcome	Avoidance of anaesthetics with marked intrinsic properties	Sample size calculation	Statement of compliance with regulatory requirements	Statement regarding possible conflict of interest
Asano 2022	Y	Y	N	N	N	Y	N	Y	Y
Brown 2011	Y	N	N	N	N	N	N	Y	Y
Cote 2011	Y	N	N	N	N	Y	N	Y	Y
Davaa 2021	Y	Y	N	N	N	Y	N	Y	Y
Han 2014	Y	N	Y	N	N	N	N	Y	N
Ilha 2011	Y	Y	N	N	N	Y	N	Y	N
Miranda 2012	Y	N	N	Y	Y	N	Y	N	Y
Li 2013	Y	Y	Y	N	Y	Y	N	Y	Y
Loy 2018	Y	N	N	N	Y	Y	N	Y	Y
Macias 2009	Y	Y	N	N	N	Y	N	Y	N
Marques 2018	Y	Y	Y	Y	Y	N	N	Y	Y
Multon 2003	Y	Y	N	N	Y	N	N	Y	N
Oh 2009	Y	Y	N	N	Y	N	N	Y	Y
Sandrow-Feinberg 2009	Y	N	N	N	N	N	N	Y	Y
Wang 2015	Y	Y	Y	Y	Y	N	N	Y	Y
Zhan 2023	Y	N	Y	N	Y	U	N	Y	Y
Zhao 2020	Y	Y	Y	N	Y	U	N	Y	Y

Y = quality criteria met. N = quality criteria not met. U = unclear if quality criteria met

Overall, the studies had mixed methodological quality, ranging from 3 to 7/9. Three studies had high methodological quality with 7 out of 9 items positively reported [36, 38, 47]. There was high compliance with regulatory requirements (16/17 studies), however, randomisation and allocation concealment were poorly documented, at 6/17 and 3/17 respectively. One study reported use of sample size calculation [45], although there remains minimal guidance on calculating sample size in animal studies [33].

### Risk of bias assessment

Studies were assessed for risk of bias using the SYRCLE risk of bias tool adapted from Cochranes’ risk of bias tool [32] (Table 3).

**Table 3:**
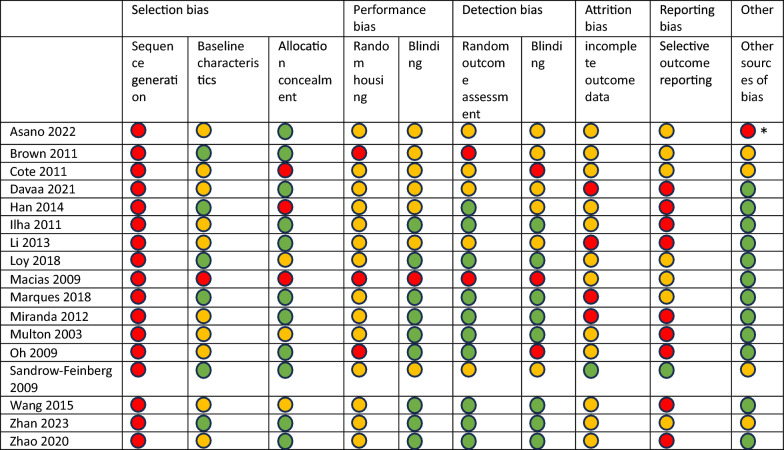
Risk of bias assessment using SYRCLE’s risk of bias tool

Bias not managed.  Bias managed.  Unclear.

*No antibiotic, analgesics or anti-inflammatory drugs were given

Blinding of the researchers and assessors was often reported, however, no study reported sequence generation as part of randomisation or randomly housing the animals, with minimal reporting on exclusions or attrition. Overall, the risk of bias was moderate to high due to lack of sufficient details to assess if confounding factors had been managed, thereby reducing confidence in the findings.

### Pathophysiological outcomes

Pathophysiological outcomes following mobilisation initiated during the first 14 days following SCI were reported in seven studies; six using BDNF [35–39] (Table 4) and one using microglial numbers [22].

**Table 4:** The effect of early mobilisation on BDNF

Study	Spinal segment examined	Unit of measure	BDNF	
			Intervention	Control
Cote 2011	T10-T11	Density (ratio to control)	1	
	T12-L1		1	
	L2-L3		1.25–2*	
	L4-L6		1.5*	
Macias 2009	L3-4	Density of BDNF IR fibres	30	15
Marques 2018 (a) (b)	T8-T10	BDNF expression (relative to control %)	125% 200%	
Sandrow-Feinberg 2009	C4 (C3 & C5)	Ratio to control	7	
Wang 2015	L5-S1	BDNF immunoreactivity	1.5*	
Zhan 2023 (a) (b) (c)	C5	Western blot technique expressed as relative to protein expression	0.75 1.25 1.25	0.5 0.5 0.5

^*^Statistically significant. BDNF (Brain-derived neurotrophic factor)

BDNF levels were greater in the groups that initiated mobilisation within 14 days of SCI compared to those that did not. For example, Zhan et al. [37] found elevated expression of BDNF at 0.75–1.25 in the groups who mobilised at low, moderate, and high intensity initiated at 7 days following injury, compared to 0.5 in the control group who did not exercise [37]. However, there was a lack of standardisation in how BDNF was collected, measured, and reported, making meta-analysis unfeasible. Mobilisation was initiated at day 5 or 7 in all studies. Marques et al. [36] included an additional group who initiated mobilisation at 14-days and had the greatest increase in BDNF levels (200% BDNF expression compared with 125% in the 7-day group) [36].

Asano et al. [22] reported on microglial numbers, concluding that early exercise (initiated day-2) promoted the reduction of the pro-inflammatory M1 microglial/ macrophages and increased the anti-inflammatory M2 microglial/ macrophages compared with those that did not exercise, suggesting that exercise during the first 14 days post SCI may be beneficial to recovery by suppressing neuroinflammation in the injured tissues [22].

### Functional outcomes

Functional outcomes following mobilisation initiated during the first 14 days following SCI were reported using the Basso, Beattie, Bresnahan (BBB) locomotor rating scale (13 studies) [22, 36, 38–42, 44–49] and the Ladder Rung Walking Task scale (6 studies) [36, 37, 39, 41, 43, 49]. Outcomes are reported at 4 weeks following SCI. Five authors did not report total group data and instead presented findings as multiple groups within their studies [36, 37, 40, 44, 47]. These outcomes have been presented and analysed as multiple groups to maintain detail and clarity. This can be found in Additional file 3.

Most groups reported improved outcomes in those who mobilised compared to those who did not. However, in three studies, the control group reported greater functional gains than the groups that exercised [36, 40, 47].

**Fig. 2:**
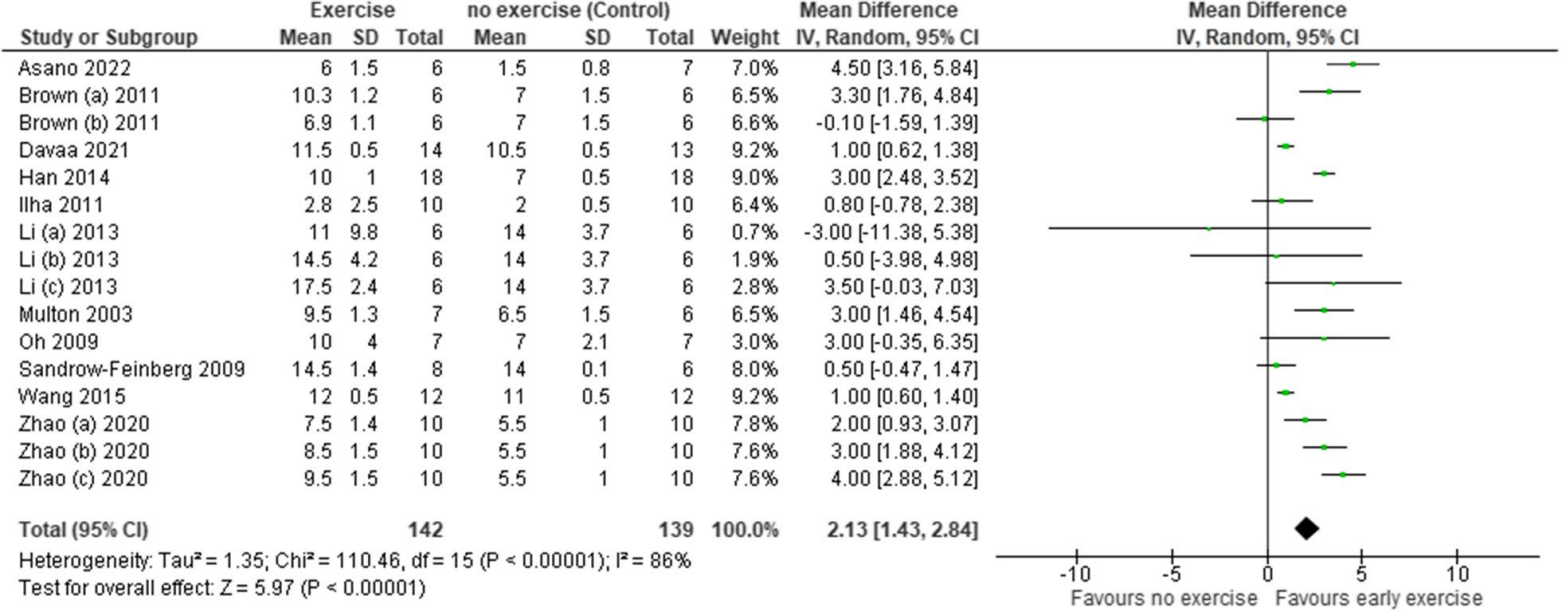
A forest plot of the effect of early mobilisation on functional outcomes using the BBB scale

**Fig.3:**
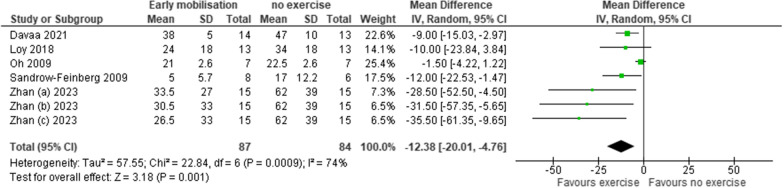
A forest plot of the effect of early mobilisation on functional outcomes using the Ladder rung walking task scale

Meta-analysis was possible in both functional outcomes (Figs. 2 and 3). Findings have been presented as multiple groups within individual studies if reported as such by the authors. Greater functional outcomes were associated with those mobilising within 14 days of SCI compared to those that did not. The mean differences were small but statistically significantly different with BBB = 2.13 (CI 1.43, 2.84) and Ladder = − 12.38 (CI − 20.01, − 4.76) (BBB p < 0.001; Ladder p = 0.001). Considerable heterogeneity was identified across the studies (BBB I^2^ = 86%; Ladder I^2^ = 74%).

#### Sub-group analysis

Sub-group meta-analysis was conducted based on timing and intensity of intervention.

##### Timing of intervention

**Fig. 4:**
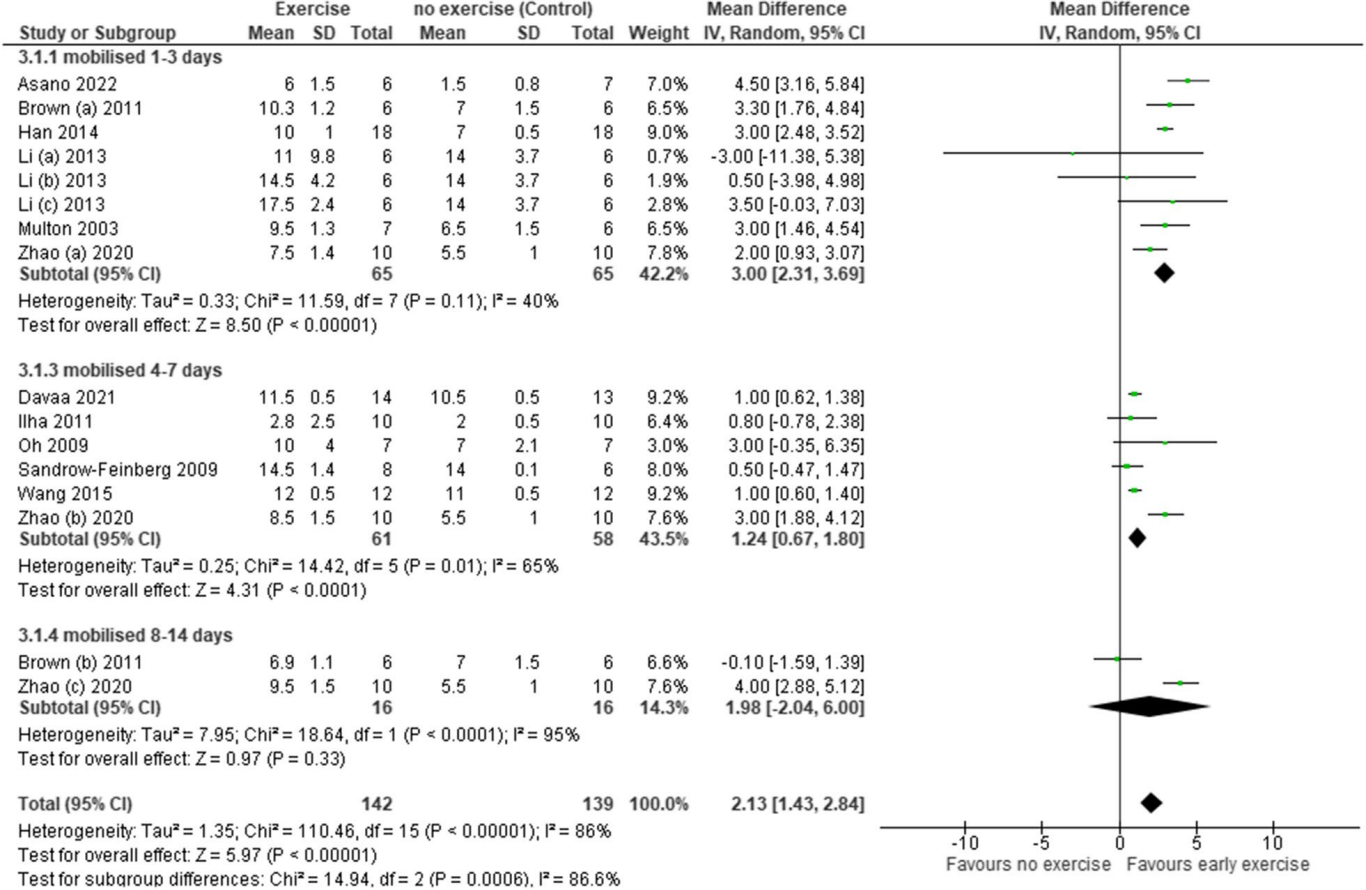
A forest plot of the effect of time mobilisation is initiated on functional outcomes using the BBB scale

A sub-group analysis was undertaken exploring outcomes following three different time periods that mobilisation was initiated: 1–3 days, 4–7 days and 8–14 days following SCI. Sufficient data for meta-analysis was available across these categories using the BBB score only (Fig. 4).

Sub-group analysis showed that greater functional gains were associated with those that initiated mobilisation between 1 and 3 days following SCI. Differences were small but statistically significant for both the 1–3-day (BBB = 3.00, CI 2.31, 3.69, p < 0.001) and 4–7-day groups (BBB = 1.24, CI 0.67, 1.80, p < 0.001). The effect of initiating mobilisation between day 8–14 was not statistically significant (BBB = 1.98, CI − 2.04, 6.00, p = 0.32) and with only two studies there was considerable heterogeneity identified (I^2^ = 95%).

##### Intensity of intervention

**Fig. 5:**
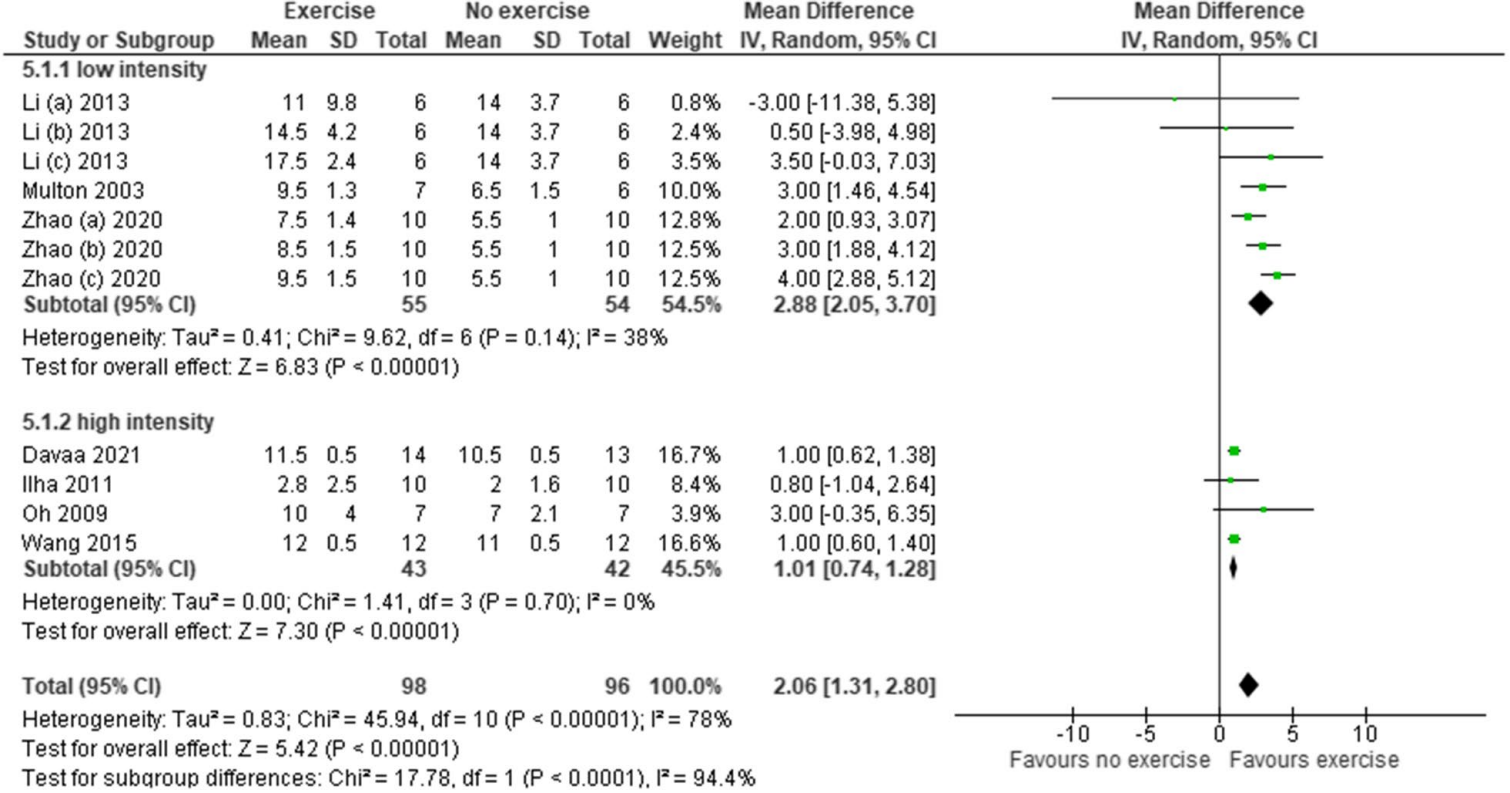
A forest plot of the effect of intensity of intervention on functional outcomes using the BBB scale

Whilst duration and frequency of interventions were unable to be included in meta-analysis, they were comparable across the studies (Table 1). There was a greater variation in the intensity of treatment and meta-analysis was possible using the BBB score only (Fig. 5.). Studies were categorised according to intensity; for the purposes of this study, those running at < 5 m/min were considered low intensity and those running at ≥ 5 m/min were considered high intensity. Six studies did not report the intensity of treatment [22, 34, 40, 42, 43, 45]. Three studies reported gradually increasing the intensity as the animal could tolerate [38, 39, 41]; for 1 study this ranged from 3.5 to 14 m/min and could therefore not be used for this analysis [39].

Greater functional gains were associated with those that mobilised at low intensity (BBB = 2.88, CI 2.05, 3.70) in comparison with those that mobilised at high intensity (BBB = 1.01, CI 0.74, 1.28). The mean difference was small but statistically significant (low intensity p < 0.001, high intensity p < 0.001) Additional file 3.

### Certainty assessment

A certainty assessment was performed using the GRADE assessment method considering the findings from the review [29, 33] (Table 5.). Certainty in the results can be downgraded from ‘high’ to ‘moderate’, ‘low’ or ‘very low’ in the presence of the criteria listed. Overall, the assessment concluded low-moderate certainty in the findings.

**Table 5:** GRADE assessment of evidence

GRADE criteria	Examples	Relevance to included studies	Outcome
Imprecision	Insufficient statistical power Wide confidence intervals Small no. of studies	Wide confidence intervals Small sample sizes No sample size calculations	Low
Inconsistency	Results not similar across studies Assessed using I^2^	I^2^ indicated considerable heterogeneity Heterogeneity unexplained Some contrasting findings	Low
Risk of bias	Limitations in study design or conduct Quality of evidence	Use of methods with controls Poor reporting of randomisation or attrition Lack of information Mixed quality across studies	Low
Indirectness	Evidence differs from the study’s intended population or outcomes	Populations of rats and mice only Outcomes reported using desired outcome measures Majority of studies addressed PICO question	Moderate
Publication bias	Lack of studies Selective publication	Most studies with small sample sizes and mixed outcomes Conflicts of interest well reported	Moderate

Grade can be assessed as high, moderate, low, or very low

## Discussion

### Summary of main findings

This review evaluated the pre-clinical evidence on the effect of early mobilisation (< 14 days) on pathophysiological responses and function in animals (rats and mice) following SCI. Early mobilisation is associated with higher levels of BDNF and lower levels of neuroinflammation, which would expect to lead to greater functional recovery. This is consistent with the outcomes of the functional measures where greater gains were also reported in animals that initiated mobilisation within 14 days of SCI compared to those who did not. Specifically, statistically significant functional gains were associated with mobilisation initiated within 3 days of SCI, or when delivered at low intensity. Overall, the level of certainty of these findings was low to moderate due to the risk of bias, mixed methodological quality, and high heterogeneity across studies.

### Comparison to previous literature

Previous pre-clinical studies have investigated the effects of mobilisation following SCI but often with small sample sizes and with variability in animal model, intervention type, timing and dose, and outcome measurement, making it difficult to draw firm conclusions [3, 18]. Increased levels of BDNF and greater function are reported following mobilisation initiated within 14-days of SCI, consistent with the outcomes of this review [3, 18, 50, 51]. However, there are few reports of worsening locomotion in those initiating mobilisation on days 1–3 [3, 18, 50–55]. This review reported on studies with varied outcomes, although, the meta-analysis concluded greatest gains when mobilisation was initiated during days 1–3. The lack of standardised measurement of BDNF prevented use of meta-analysis for more robust investigation in this field.

Pre-clinical and clinical literature have reported wide variation in training dose, with a lack of consensus in the optimum intensity of mobilisation following SCI [5, 9, 56]. Worsening function or limited recovery have been reported in pre-clinical studies following high intensity treadmill training, although definitions of low or high intensity remain subjective [18, 51, 53]. Within this review, contrasting outcomes were reported across studies, but meta-analysis of seven studies also concluded greater gains following low compared with high intensity training.

### Strengths and limitations

#### Use of pre-clinical studies in a systematic review

The use of systematic review methods, including meta-analysis and PRISMA reporting, were a robust way of investigating and consolidating the uncertain and apparent conflicting outcomes of early mobilisation following SCI [20, 29]. However, as with all systematic reviews, it is possible that not all relevant studies were identified. Use of pre-clinical studies had the advantage of investigating outcomes of early mobilisation using robust methods with control groups, not commonly seen in clinical trials in SCI due to ethical challenges, thereby overcoming the impact of spontaneous recovery. However, most animals in the control groups were not immobilised (consistent with clinical practice) but allowed some freedom of movement; this was often difficult to establish due to limited reporting [6, 9].

#### Exploration of heterogeneity

The complexity of synthesising pre-clinical studies is recognised as a challenge in undertaking pre-clinical systematic reviews [20]. Whilst attempts were made to reduce variables, it is recognised that those which remained may have contributed to the high heterogeneity identified across the studies. Rats and mice are commonly reported to be comparable to each other and appeared consistent with the outcomes of this review, there remains the possibility of variability in pathophysiological and functional responses across and within species and genders [25, 28, 57].

Severity and level of spinal injury influence patterns of recovery [58]. Most studies reported on thoracic injury induced by contusion, although outcomes from cervical injuries and transections were also included, with severity not easily ascertained. The pre-clinical literature lacked clarity on the influence of mode of injury on outcomes. This led to the decision to include all types of SCI, which may have added to the heterogeneity of the sample and is a possible limitation of the study [25, 28].

#### Measurement of outcomes

Pathophysiological outcomes were measured through identification of BDNF, a commonly used indicator of neuro recovery across animals and humans, although it’s specific role in recovery following injury is uncertain [18, 25, 59]. Studies also lacked standardisation in measurement and reporting of BDNF, making it difficult to consolidate or compare findings across the studies. The BBB scale is regarded as a standardised and reliable functional measure for use in rats, also widely used in mice, however, clinically significant change scores are yet to be determined, nor a clear understanding of how this translates to functional change in humans [23, 58].

#### Methodological quality

A significant limitation of this review was the methodological quality in the included studies, with only two assessed as ‘high’. Reporting of sample size calculation, allocation concealment and randomisation were often lacking, with inconsistent use of blinded assessment, leading to increased risk of bias and caution in the interpretation of the findings [30, 33]. This may have influenced the heterogeneity identified across these studies in meta-analysis.

#### Considerations for transferability

Protocol decisions were made with the aim of maximising transferability of results for consideration in clinical trials or practice, with murids chosen for their potential transferability of outcomes of motor function following SCI [28, 57]. This review reported on a slightly lower ratio of male to female participants than is typically reported in human SCI studies (M:F = 3:2, compared to 7:3) [5, 60]. Whilst gender has been reported not to be a predictor of clinical outcomes, the influence of gender on pre-clinical outcomes remains inconclusive [5, 25, 57, 61]. Most studies reported outcomes following SCI induced by contusion, regarded as the closest to human SCI models and therefore the most clinically relevant, although these were predominantly at a thoracic level, known to be chosen for experimental convenience over clinical relevance, thereby potentially reducing transferability [3, 24, 25].

In addition, this review reported on short term outcomes only. Whilst this could be considered a limitation, it highlights the ethical implications associated with this type of research and why there are significant gaps in the evidence base for early mobilisation [30].

### Clinical implications

When clinical studies are difficult to undertake due to practical and ethical challenges, pre-clinical studies have a role in gathering early evidence of interventions [18, 20, 33]. Outcomes from this review suggest that early mobilisation (< 14 days) in rats and mice and may promote further functional recovery in the short term. This indicates that delaying mobilisation may lead to adverse pathophysiological outcomes and potential worse functional recovery. Outcomes suggest mobilisation could be considered within three days of injury, particularly if delivered at low intensity, although further clarification is needed on how this is defined [20, 33].

### Unanswered questions and future research

Whilst the findings from this study suggest the potential for greater functional gains following early mobilisation post SCI, certainty in these findings was moderate to low. Further robust animal-model studies are needed to establish the optimum time and intensity of mobilisation to reduce risk of bias, gain further precision, and increase reliability in outcomes. This could be enhanced by increased methodological quality in pre-clinical studies to include randomisation and reporting of sample size calculations, in addition to greater standardisation in the measurement and reporting of measures of pathophysiology.

It remains unknown whether the outcomes from this study would be replicated in patients with SCI, nor maintained over the longer term in either animal or human models. Further clinical studies are required to continue to build knowledge in this area.

## Conclusion

Early mobilisation, instigated within 14-days of SCI, may be an effective way of promoting recovery and improving functional outcomes in animal models, with delays harmful to recovery. This adds to the knowledge and understanding of the potential risks and benefits associated with early mobilisation. Identification of the optimum intensity and time to initiate mobilisation remains challenging, with outcomes from this study supporting further research in this field to guide future clinical practice.

### Supplementary Information

**Additional file 1.** Example search strategy.

**Additional file 2.** Excluded studies.

**Additional file 3.** Outcomes of function.

## Data Availability

I don’t have any research data outside the submitted manuscript file.
