# Supplementary material for: The effect of early mobilisation (< 14 days) on pathophysiological and functional outcomes in animals with induced spinal cord injury: a systematic review with meta-analysis
Source: BMC Neurosci. 2024 Mar 25;25:20. doi: 10.1186/s12868-024-00862-3 (PMC10964644; doi:10.1186/s12868-024-00862-3)
Supplement: Supplementary file 1 — Additional file 1. Example search strategy. [file 12868_2024_862_MOESM1_ESM.docx]

### Example search strategy

| **Concept** | **Search term** |
| --- | --- |
| 1.Spinal cord injury | **(((((((((ALL=(spinal cord injur*))) OR ALL=(spinal cord contusion)) OR ALL=(spinal cord hemisection)) OR ALL=(spinal cord transection)) OR ALL=(spinal cord compression)) OR ALL=(tetrapleg*)) OR ALL=(quadripleg*)) OR ALL=(paraplegi*)) OR ALL=(TI Spin*)** |
| 2.Mobilisation | **((ALL=(acute physical intervention$)) OR AB=(locomotor training)) OR AB=(neuro-rehabilitation) OR (((((((ALL=(early mobili?ation))) OR ALL=(mobili?sation)) OR ALL=(early ambulation)) OR ALL=(Accelerated Ambulation)) OR ALL=(early Rehabilitation)) OR ALL=(Early Activit*)) OR ALL=(Early Exercise) OR ALL=(treadmill training)** |
| 3. Animal models | **((((((((((ALL=(animal models)) OR ALL=(pre-clinical)) OR ALL=(rodentia)) OR ALL=(Rat*)) OR ALL=(mice)) OR TI=(mus)) OR AB=(mouse)) OR AB=(murine)) OR TI=(murine))** |
| 4.Pathophysiology | **(((ALL=(PATHOLOG*)) OR ALL=(PHYSIOLOG*)) OR ALL=(PATHOPHYSIOLOGY)) OR ALL=(Physiopathological)** |
| 5. Function | **'Basso, Beattie and Bresnahan'** **(All Fields) (ladder test) (All Fields) OR** **(ladder rung test)** **(All Fields) OR**  **(horizontal ladder test)** **(All Fields) OR** **(ladder scores)** **(All Fields)** |
| 1 AND 2 AND 3 AND (4 OR 5) | |
