# Supplementary material for: The effect of early mobilisation (< 14 days) on pathophysiological and functional outcomes in animals with induced spinal cord injury: a systematic review with meta-analysis
Source: BMC Neurosci. 2024 Mar 25;25:20. doi: 10.1186/s12868-024-00862-3 (PMC10964644; doi:10.1186/s12868-024-00862-3)
Supplement: Supplementary file 2 — Additional file 2. Excluded studies. [file 12868_2024_862_MOESM2_ESM.docx]

## Excluded studies (15)

| Reason for exclusion | Study |
| --- | --- |
| Incorrect outcome domain or outcome measures (11) | Chopek 15[1] |
|  | Dupont 1999[2] |
|  | Endo 2009[3] |
|  | Engesser 2005[4] |
|  | Goldshmit 2008[5] |
|  | Keeler 2012[6] |
|  | Kiss 2022[7] |
|  | Liu 2010[8] |
|  | Liu 2012 [9] |
|  | Martinez 2012[10] |
|  | Ying 2005[11] |
| Combined interventions (1) | Sun 2013[12] |
| Injected with BDNF inhibitor (1) | Ying 2008[13] |
| Conference abstract (1) | Benton 2009 [14] |
| No spinal injury (1) | Perreau 2005[15] |

1. Chopek, J.W., et al., *Serotonin receptor and KCC2 gene expression in lumbar flexor and extensor motoneurons posttransection with and without passive cycling.* J Neurophysiol, 2015. **113**(5): p. 1369-76.

2. Dupont-Versteegden, E.M., R. Houle, J. Gurley, C. Peterson, C., *Activated satellite cells fail to restore myonuclear number in spinal cord transected and exercised rats.* Cell Physiology, 1999. **46**: p. C589-C597.

3. Endo, T., et al., *Early exercise in spinal cord injured rats induces allodynia through TrkB signaling.* Biochem Biophys Res Commun, 2009. **381**(3): p. 339-44.

4. Engesser-Cesar C, A.A., Basso DM, Edgerton VR, Cotman CW., *Voluntary wheel running improves recovery from a moderate spinal cord injury.* J Neurotrauma, 2005. **22**: p. 157-71.

5. Goldshmit, Y., et al., *Treadmill training after spinal cord hemisection in mice promotes axonal sprouting and synapse formation and improves motor recovery.* J Neurotrauma, 2008. **25**(5): p. 449-65.

6. Keeler, B.E., et al., *Acute and prolonged hindlimb exercise elicits different gene expression in motoneurons than sensory neurons after spinal cord injury.* Brain Res, 2012. **1438**: p. 8-21.

7. Kiss Bimbova, K., et al., *Activation of Three Major Signaling Pathways After Endurance Training and Spinal Cord Injury.* Mol Neurobiol, 2022. **59**(2): p. 950-967.

8. Liu, G., et al., *Cycling exercise affects the expression of apoptosis-associated microRNAs after spinal cord injury in rats.* Exp Neurol, 2010. **226**(1): p. 200-6.

9. Liu, G., et al., *Exercise modulates microRNAs that affect the PTEN/mTOR pathway in rats after spinal cord injury.* Exp Neurol, 2012. **233**(1): p. 447-56.

10. Martinez, M., et al., *Effect of locomotor training in completely spinalized cats previously submitted to a spinal hemisection.* J Neurosci, 2012. **32**(32): p. 10961-70.

11. Ying, Z., et al., *Exercise restores levels of neurotrophins and synaptic plasticity following spinal cord injury.* Exp Neurol, 2005. **193**(2): p. 411-9.

12. Sun, T., et al., *Cotransplantation of olfactory ensheathing cells and Schwann cells combined with treadmill training promotes functional recovery in rats with contused spinal cords.* Cell Transplantation, 2013. **22 Suppl 1**: p. S27-38.

13. Ying, Z., et al., *BDNF-exercise interactions in the recovery of symmetrical stepping after a cervical hemisection in rats.* Neuroscience, 2008. **155**(4): p. 1070-8.

14. Benton, R.M., M. Brown, E. Shum-Siu, A. Whelan, A. Magnuson, D., *Analysis of gene expression associated with microvascular activation and neuroplasticity in acute activity-based rehabilitation following traumatic rat spinal cord injury (SCI)*, in *13th International Symposium on Neural Regeneration (ISNR)*. 2009, Neurorehabilitation and Neural Repair: Pacific Grove, CA. p. 972.

15. Perreau, V.A., P. Anderson, A. Cotman, C., *Exercise-Induced Gene Expression Changes in the Rat Spinal Cord.* Gene Expression, 2005. **12**: p. 107-121.
