# Supplementary material for: The effect of early mobilisation (< 14 days) on pathophysiological and functional outcomes in animals with induced spinal cord injury: a systematic review with meta-analysis
Source: BMC Neurosci. 2024 Mar 25;25:20. doi: 10.1186/s12868-024-00862-3 (PMC10964644; doi:10.1186/s12868-024-00862-3)
Supplement: Supplementary file 3 — Additional file 3. Outcomes of function. [file 12868_2024_862_MOESM3_ESM.docx]

### Outcomes of function

| **Study** | **Mean BBB score (SD)** | | **Mean Ladder score (SD)** | |
| --- | --- | --- | --- | --- |
|  | **Intervention** | **Control** | **Intervention** | **Control** |
| Asano 2022 | 6 ±1.5 | 1.5 ±0.8 | - | - |
| Brown 2011 (a) | 10.3 ±1.2 | 7 ±1.5 | - | - |
| Brown 2011 (b) | 6.9 ±1.1 | 7 ±1.5 | - | - |
| Davaa 2021 | 11.5 ±0.5 | 10.5 ±0.5 | 38 ±5 | 47 ±10 |
| Han 2014 | 10 ±1.0 | 7 ±0.5 | - | - |
| Ilha 2011 | 2.8 ±2.5 | 2 ±1.6 | - | - |
| Li 2013 (a) | 11 ±9.8 | 14 ±3.7 | - | - |
| Li 2013 (b) | 14.5 ±4.2 | 14 ±3.7 | - | - |
| Li 2013 (c) | 17.5 ±2.4 | 14 ±3.7 | - | - |
| Loy 2018 | - | - | 24 ±18 | 34 ±18 |
| Marques 2018 (a) | 8 (NR) | 11 (NR) | 86 (NR) | 75 (NR) |
| Marques 2018 (b) | 12 (NR) | 11 (NR) | 72 (NR) | 75 (NR) |
| Miranda 2012 | 8.5 (NR) | 6.5 (NR) | - | - |
| Multon 2003 | 9.5 ±1.3 | 6.5 ±1.5 | - | - |
| Oh 2009 | 10 ±4 | 7 ±2.1 | 21 ±2.6 | 22.5 ±2.6 |
| Sandrow-Feinberg 2009 | 14.5 ±1.4 | 14 ±0 | 5 ±5.7 | 17 ±12.2 |
| Wang 2015 | 12 ±0.5 | 11 ±0.5 | - | - |
| Zhan 2023 (a) | - | - | 33.5 ±27 | 62 ±39 |
| Zhan 2023 (b) | - | - | 30.5 ±33 | 62 ±39 |
| Zhan 2023 (c) | - | - | 26.5 ±33 | 62 ±39 |
| Zhao 2020 (a) | 7.5 ±1.4 | 5.5 ±1.0 | - | - |
| Zhao 2020 (b) | 8.5 ±1.5 | 5.5 ±1.0 | - | - |
| Zhao 2020 (c) | 9.5 ±1.5 | 5.5 ±1.0 | - | - |

NR-Not reported. Ladder scores are represented as % errors (Functional improvements were identified by a lower percentage of errors)
